# A review of teleradiology in Africa – Towards mobile teleradiology in Nigeria

**DOI:** 10.4102/sajr.v26i1.2257

**Published:** 2022-01-11

**Authors:** Mohammed Y. Tahir, Maurice Mars, Richard E. Scott

**Affiliations:** 1 Department of TeleHealth, College of Health Sciences, School of Nursing and Public Health, University of KwaZulu-Natal, Durban, South Africa; 2 Medical Imaging Informatics ISID, King Abdulaziz Medical City, Jeddah, Saudi Arabia; 3 Department of Community Health Sciences, Cumming School of Medicine, University of Calgary, Calgary, Canada

**Keywords:** teleradiology, mobile teleradiology, cellphone, Nigeria, developing country

## Abstract

eHealth is promoted as a means to strengthen health systems and facilitate universal health coverage. Sub-components (e.g. telehealth, telemedicine, mhealth) are seen as mitigators of healthcare provider shortages and poor rural and remote access. Teleradiology (including mobile teleradiology), widespread in developed nations, is uncommon in developing nations. Decision- and policy-makers require evidence to inform their decisions regarding implementation of mobile teleradiology in Nigeria and other sub-Saharan countries. To gather evidence, Scopus and PubMed were searched using defined search strings (September 2020). Duplicates were removed, and titles and abstracts reviewed using specified selection criteria. Full-text papers of selected resources were retrieved and reviewed against the criteria. Insight from included studies was charted for eight *a priori* categories of information: needs assessment, implementation, connectivity, evaluation, costing, image display, image capture and concordance. Fifty-seven articles were identified, duplicates removed and titles and abstracts of remaining articles reviewed against study criteria. Twenty-six papers remained. After review of full-texts, ten met the study criteria. These were summarised, and key insights for the eight categories were charted. Few papers have been published on teleradiology in sub-Saharan Africa. Teleradiology, including mobile teleradiology, is feasible in sub-Saharan Africa for routine X-ray support of patients and healthcare providers in rural and remote locations. Former technical issues (image quality, transmission speed, image compression) have been largely obviated through the high-speed, high-resolution digital imaging and network transmission capabilities of contemporary smartphones and mobile networks, where accessible. Comprehensive studies within the region are needed to guide the widespread introduction of mobile teleradiology.

## Introduction

eHealth, the cost-effective and secure use of information and communication technologies (ICTs) for health and health-related fields,^1^ can contribute to health systems’ strengthening in several ways. It can improve the availability, quality and use of information and evidence through strengthened health information systems and public health surveillance systems, as well as increase access to healthcare services by reducing distance and time barriers through telemedicine. This is particularly beneficial for rural and underserved communities in developing countries – groups that traditionally suffer from a lack of access to healthcare.^2^

The term eHealth, more recently Digital Health, is an umbrella term that covers a variety of activities such as:

e-commerce (the business side), e-learning (the training – awareness, teaching, instruction, and education – side), health informatics (the data gathering, storage, analysis, and distribution side), and telehealth including telemedicine (the interactive – real-time or store-and-forward – side).^3^

More recent applications such as mHealth, the use of mobile devices such as cell phones, can incorporate elements of each.^2^

One such use is teleradiology. The benefits of teleradiology include earlier diagnosis, leading to quicker treatment and better patient care, and ultimately less burden on the healthcare system, plus less travel and reduced expense for the mainly poverty stricken, rural population and especially for the chronically ill.^4^ However, conventional radiology is still common in the developing world. The evolution of conventional radiology to digital radiology and then to teleradiology has also been described.^4^

With the advent of the Internet, www, smartphones and cellular networks, teleradiology and mobile teleradiology have emerged. Teleradiology is the electronic capture (using digital detectors or digital photography of X-ray films), transmission (from one location to another), storage (often within erecords) and retrieval and display (on monitors) of radiological patient images. Mobile teleradiology is effectively teleradiology facilitated through the use of mobile devices, primarily smartphones, although related approaches using instant messaging (e.g. WhatsApp) are becoming popular.^5^ Regardless, these approaches allow for simultaneous sharing of images with other clinicians in other locations, obviating lost images and facilitating remote interpretation and consultation. If not ubiquitous, teleradiology is at least considered ‘widespread throughout modern radiology practice’^6^ and to be amongst the most established and widely used telemedicine specialties.^4^

Nigeria, located in West Africa, is the most populous country in Africa and seventh in the world with a population of 206.1 million in 2020 spread across 923 768 km^2^,^7^ with 48.8% of people living in rural areas in 2019.^8^ Health indicators in Nigeria are some of the worst in Africa. The country has faced constant health threats because of civil unrest and a lack of adequate funding, deteriorating healthcare facilities, concentration of healthcare providers and services in urban centres, poor patient access to healthcare and inadequate numbers of healthcare providers.^2,9^ Private healthcare providers have filled some gaps and account for up to 70% of health services coverage in Nigeria, with limited involvement of non-government organisations (NGOs).^9^ Indeed, the sub-Saharan Africa (SSA) region averages fewer than 22 doctors per 100 000 people, and 14 countries within the region do not have a single radiologist, whilst Nigeria has just 250–300 or about one radiologist for every 600 000 people.^2,9^ Nigeria has one of the fastest growing populations globally, estimated to reach 440 million people by 2050. The country’s healthcare system is organised into primary, secondary and tertiary healthcare levels.^9^ The local government areas (LGAs) are responsible for primary healthcare, the State Governments are responsible for providing secondary care and the Federal Government is responsible for policy development, regulation, overall stewardship and providing tertiary care.^9,10^

This pre-existing situation is now compounded by the impact of coronavirus disease 2019 (COVID-19), with Nigeria being one of the first nine African countries reporting cases by the first week of March 2020.^11^ Similar to many countries, the pandemic has caused greater socioeconomic hardship, further stretching of its ill equipped and underprepared healthcare system, rising mis- and dis-information negatively impacting behaviour and impaired access to essential medicines potentially worsening chronic conditions.^12,13,14^

Amidst this disarray, practical solutions to address known health needs are required. In Nigeria, because of the scarcity of radiologists, many imaging investigations are performed and interpreted by hospital medical officers or perhaps radiographers.^15^ For example, at Zambuk General Hospital in Zambuk, Gombe State (north-eastern Nigeria, ~450 km from Abuja), when a medical officer requests an X-ray study, the patient is referred to the radiology department. There, they will be charged a fee for the imaging procedure only. After the X-ray study is completed, the patient takes the films back to the medical officer who then decides whether or not a radiologist’s report is needed. If a report is required, the patient must make up to a 100 km round trip to the State capital, Gombe city, for the film to be reported and pay a fee for the report.

Teleradiology would avoid patient travel, reduce out of pocket travel expenses, provide more rapid diagnosis and improve diagnostic accuracy and reliability. This may also reduce patient transfer, re-hospitalisation and length of in-hospital stay.

Although teleradiology has been used in the developing world, it remains relatively rare in Nigeria.^2,9^ Given the proven benefits of teleradiology, and the scarcity and maldistribution of radiologists, growing populations, the challenges of poor health and the still developing technological infrastructure in developing countries, is teleradiology, particularly mobile teleradiology, a feasible and suitable solution, in particular for Nigeria? Whilst many clinical modalities of radiology exist (e.g. ultrasound [US], computerised tomography [CT], magnetic resonance imaging [MRI]), this paper is focused on mobile teleradiology for routine X-rays within SSA. The aim of this study was to review the literature on contemporary applications of teleradiology in African countries and to provide insight and perspective upon which to base informed decisions on if and how mobile teleradiology could be implemented in Nigeria.

## Methods

For this scoping review, a series of focused searches of Scopus and PubMed were performed in September 2020 (M.Y.T. and M.M.). The PubMed search string was (‘radiology’ [MeSH] AND ‘telemedicine’ [MeSH]) OR teleradiology [All Fields]) OR ‘mobile teleradiology’ OR ‘mobile tele-radiology’ OR (‘radiology’ [MeSH] AND [mhealth OR m-health OR ‘mobile health’]) AND ‘Africa’ [MeSH], and in Scopus, the following search string was used (TITLE-ABS-KEY [radiology AND telemedicine] OR teleradiology OR ‘mobile teleradiology’ OR ‘mobile tele-radiology’) OR TITLE-ABS-KEY (radiology AND [mhealth OR m-health OR ‘mobile health’]) AND TITLE-ABS-KEY [Africa]).

Duplicates were removed. All authors then reviewed titles and abstracts applying the following inclusion criteria: English language, resource addressed use of teleradiology in Africa. Papers were excluded if they were published before 2009 (to ensure currency in this rapidly evolving area) or primarily addressed more sophisticated imaging approaches: picture archiving and communication system (PACS), CT, MRI, US or Nuclear Medicine (NM). Any disagreements were resolved by consensus.

Full papers of remaining resources were retrieved, and each paper again reviewed applying the inclusion and exclusion criteria, with disagreements resolved by consensus. Data from the final selected resources were charted into an Excel spreadsheet (Microsoft^®^ Excel^®^; Redmond, Washington, United States) by two authors (M.Y.T. and R.E.S.) and cross-checked. Charted data included citation details (authors, title, year), country or region and insight provided regarding eight *a priori* categories: needs assessment, implementation, connectivity, evaluation, costing, image display, image capture and concordance.

### Ethical considerations

Approval was provided by the Humanities and Social Sciences Research Ethics Committee, University of KwaZulu-Natal; Protocol Reference Number – HSS/0508/017D.

## Results

**FIGURE 1:**
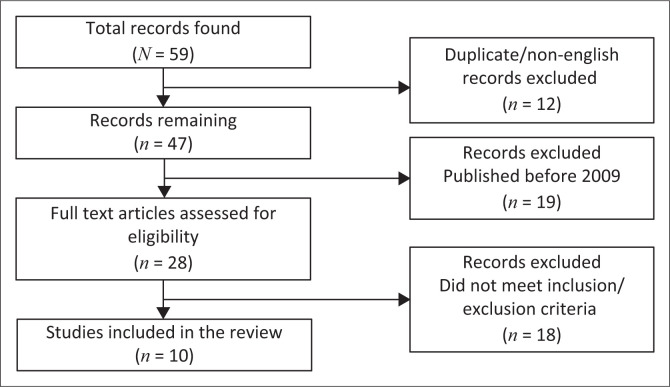
Preferred Reporting Items for Systematic Reviews and Meta-Analyses (PRISMA) flowchart for literature search.

A total of 59 articles were generated from the searches (Figure 1). Ten met the inclusion criteria for the study after a full-text review.^16,17,18,19,20,21,22,23,24,25^ These papers examined aspects of teleradiology from throughout SSA,^16,24^ regions (West Africa)^25^ or specific countries: Angola,^19^ Ethiopia,^20^ Malawi,^21^ Mali,^18^ South Africa^17^ and Zambia.^23^ The study in one paper was performed in the United States but was intended to emulate the setting and process in Botswana and was included.^22^ Each paper was reviewed, summarised and the presence of insight regarding the eight categories noted (Table 1); the results for each category are presented below.

**TABLE 1:** Summary of each resource and presence of material relevant to the eight categories.

First author [Ref] (Year) [Country/region]	Summary	Needs assessment	Implementation	Connectivity	Evaluation	Costing	Image display	Image capture	Concordance
Andronikou et al.^17^ (2011) [sub-Saharan Africa]	Paediatric radiology in Africa. Discusses the World Health Organisation’s ‘World Health Imaging System for Radiography’ (WHIS-RAD) initiative and need for volunteer paediatric radiologists for reporting and mentoring. Highlights general issues of poverty, mortality, access to care, disease burden, HIV/AIDS, trauma, lack of human resources and lack of basic radiology equipment. Also highlights challenges with conventional radiology (e.g. need for: controlled temperature and humidity, water use, chemical use, stable power supply), plus additional paediatric-specific issues – orphans, lack of parental care and violence. Mentions online access, central data storage and PACS of Téléradiologie sans Frontières. Suggests task shifting to ameliorate staff shortages, and that digital imaging may offer advantages – obviating film development, increasing access to remote experts for consultation.	✓	✓	-	-	✓	✓	✓	-
Coulborn et al.^21^ (2012) [Malawi]	Médecins Sans Frontières initiative for remote teleradiology image interpretation and diagnosis for one district to aid TB screening. Notes creation of a database to store request forms, images and patient summaries. Highlights poverty, access to care, lack of human resources (‘absence of radiologists in 14 SSA countries’). Provides a descriptive analysis of the teleradiology programme and patient clinical outcomes and demonstrated positive impacts in changing ‘patient management or correcting misdiagnosis improving patient morbidity and mortality’. Demonstrated ‘feasibility and utility of teleradiology’ but need for judicious implementation.	✓	✓	-	-	-	✓	✓	-
Shiferaw and Zolfo^20^ (2012) [Ethiopia]	Presents challenges, failures and successes experienced during set-up and implementation of a pilot telemedicine programme with a teleradiology component across 10 sites. Notes need for guidelines and ‘IT security protocols, consent for image capture, and storage of images and patient records in password protected systems’. Used natural light to capture images to compensate for settings with unstable power sources. Highlights influence of non-technology issues – ‘e-governance, e-readiness, enabling policies, multi-sectoral involvement, and capacity building processes’, as well as practical issues of ‘local context, training, staff turnover, simplest technology, and system integration’. It is concluded there is no single perfect eHealth technology, requiring combined solutions adjusted to local contexts. States the study demonstrated the application was feasible in resource-constrained remote and urbanised areas and showed ‘practical applicability beyond reasonable doubts’.	✓	✓	-	-	-	-	✓	-
Harris^25^ (2013) [West Africa]	Describes a personal experience of a radiologist working in a small radiology department aboard the *Africa Mercy*, ‘the largest private hospital ship in the world’, which has sustained delivery of medical and surgical care to 15 West Africa countries. Noted storage of compressed images on a secure server for teleradiology and use of electronic radiographic reports archived on the server together with scanned copies of patient paper medical records.	✓	-	-	-	-	-	✓	-
Mars^24^ (2013) [Africa]	Describes several aspects of telemedicine usage across SSA, including a brief history and comment on teleradiology. Notes difficulties related to scanning or photographing images and transmitting large image files. Highlights lack of human resources, access to care and reduced patient travel. Discusses legal, regulatory and ethical barriers for local and inter-jurisdictional practice (liability, licensure, jurisdiction, quality and continuity of care, confidentiality, data security, consent, authentication and remuneration), the need for standards and the promise of mobile phone (mhealth) use in Africa.	✓	-	-	-	-	-	-	-
Zennaro et al.^19^ (2013) [Angola]	Describes ‘evaluation’ of a digital radiology feasibility project in a single hospital to acquire, print and transmit images for remote consultation. Highlights advantages of digital imaging, details of equipment and process used and focus on paediatric remote consultation, implementation difficulties, training needs, technology issues, improvement in image quality. Notes possible legal challenges to the sustainability of programmes, compliance with European privacy regulations and use of a virtual private network for secure data transfer. Concludes: ‘The implementation of a digital X-ray device is feasible in low resource settings with significant improvement in quality of X-ray images compared to standard screen film radiology’. However, also suggests the primary role of teleradiology should be facilitating training and capacity building.	-	✓	✓	-	✓	-	-	-
Griggs et al.^16^ (2014) [South Africa]	‘Evaluates’ the practicality and sustainability of a pilot paediatric teleradiology project for volunteer –based (40 volunteers from 17 countries) remote second opinion, an initiative of the World Federation of Pediatric Imaging (WFPI) program, in a single hospital. Direct JPEG conversions of digital radiographs and request forms were e-mailed for the second opinion. Highlights contribution of radiology to diagnosis and management, lack of services, lack of human resources (absence of radiologists in some countries) and other challenges (sustainability, language, legal, technical and confidentiality). Promotes local training and capacity-building to avoid reliance on external support. Concludes ‘teleradiology is a viable option to alleviate radiologist shortages in underserved areas’.	-	✓	✓	✓	-	✓	✓	-
Schwartz et al.^22^ (2014) (United States – intended to emulate setting in Botswana)	Demonstrated the diagnostic accuracy of digital photographs of plain film chest X-rays (viewed on a light box) obtained *using a mobile phone* and sent by e-mail using a mobile phone network. Noted use of secure e-mail in Botswana. ‘Non-inferiority’ to plain film chest X-rays was shown. Highlighted human resource shortages and the constant and rapid improvement in the quality of mobile phone digital cameras.	-	-	-	✓	-	-	-	-
Sangare et al.^18^ (2015) [Mali]	Reviews the history of the initiation and development of the national teleradiology programme that connects seven public hospitals and a private clinic in Mali, noting alignment of programme growth with Internet availability. Examined improvement in accuracy of diagnosis for patients by referring doctors. Demonstrated ‘reduced professional isolation’ and an ‘increase in confidence and diagnostic ability’ of local doctors over time although with some sole or altered diagnosis by consulted radiologists. Highlighted human resource shortages, need for improved diagnosis and care, reduced travel burden for patients, long distances and poor road networks, out-of-pocket patients costs, tendency for North-South collaboration rather than local regional or national platform development and capacity building as demonstrated here and need for local ownership of the network for sustainability. Also noted the impact of conflict/war in the region. The program shifted to digital cameras for image capture over time. Transmission by broadband and VSAT (for remote locations) was used.	-	✓	✓	-	✓	-	✓	-
Strahan and McAdam^23^ (2017) [Zambia]	Discusses experience with computer radiography in Zambia to demonstrate feasibility in a remote African setting. Highlights human resource shortages (no radiologist in the country). Challenges during installation and set-up were described. Benefits accrued were noted: avoided wet film chemicals and processing, reduced radiation exposure, repeat examinations avoided, increased efficiency, lost image studies eliminated, remote (Internet) servicing, shareable images for referral, remotely accessible database of images, plus remote review and reporting (teleradiology with volunteer overseas radiologist). Noted use of fixed IP address and https: certificate for increased security and greater ease of storing images for later retrieval and education. Despite ‘success’ and benefits – replication elsewhere not planned.	-	✓	-	-	✓	-	✓	-

HIV, human immunodeficiency virus; AIDS, acquired immune deficiency syndrome; TB, tuberculosis; SSA, sub-Saharan Africa; VSAT, very small aperture terminal; Ref, reference.

✓ , Denotes material relevant to an identified category is present.

After a preliminary review of the papers, major findings under the eight categories were charted independently by two authors (M.Y.T. and R.E.S.). The findings were jointly reviewed and finalised by consensus and are reported below.

### Needs assessment

Needs assessment has been described as a systematic process for determining the gap between the current condition and the desired condition (called a need) and how to address the need and close the gap.^26^ No retrieved paper provided such a structured needs assessment although most identified human resource shortages (even an absence of radiologists in some countries), implying a self-evident need. For example, one identified teleradiology as a ‘viable option to alleviate radiologist shortages in underserved areas’.^17^

### Implementation

Implementation was described as the process of putting a decision or plan into effect, describing the step-by-step stages of execution for the plan. No paper provided such a structured description for a project or intervention programme. Simplistic descriptions of some aspects of an intervention for implementations were noted. Thus, the provision of required infrastructure (Internet access and a digital camera) was described to initiate a low-cost telemedicine service linking doctors at hospitals in the developing world with volunteer consultants in the developed world.^17^

Three papers described clearer examples of implementation^18,19,20^ although specific implementation plans were not described. The first addressed a national teleradiology programme in Mali, which required 3–6 months for implementation, undertaking local consultation, training, installation of very small aperture terminal (VSAT) antennae at three remote hospitals and testing of connectivity. The importance of local management and ownership was highlighted.^18^ The second described how the implementation of a low-cost digital X-ray device was feasible in low resource settings and included a computer system and software configuration and installation, as well as a training course for local staff in local languages.^19^ The third described an unsuccessful implementation in Ethiopia and highlighted lessons learnt related to implementation, including the importance of peripheral issues such as e-government readiness, enabling policies, multi-sectoral involvement and capacity building processes, as well as more practical issues such as understanding existing referral practice and experiences, developing guidelines and ensuring ICT security protocols are established.^20^

### Connectivity

Papers were examined to determine whether a description was provided of the mode of connection between the source of the image and a reporting station. Six papers addressed connectivity,^16,17,18,19,20,21^ with e-mail as the predominant mode using transmission through broadband, cellular networks, VSAT, asymmetric digital subscriber line (ADSL) or dial-up Internet access, either transmitted directly, to a website, or using a virtual private network (VPN).

One described the use of a slow Internet connection and downloading of images in compressed format and e-mail to send them for reporting as an attachment.^17^ Another described evolution from the use of e-mail to a web-based telemedicine service that allowed data uploading and downloading directly to and from the website with e-mail used only for creating notification alerts.^21^ A third paper examined use of JPEG radiographic images sent by e-mail as JPG files to reduce file size.^16^ Other options used in Mali included broadband connectivity or, for remote sites, VSAT.^18^ In Ethiopia, transmission of images initially used dial-up Internet access, but this frequently experienced ‘download time expired’ errors. This was subsequently resolved using open source software that collated and compressed a minimal clinical dataset for transmission.^20^ Finally, in Angola, an existing ADSL Internet connection using a secured VPN was used for file transfer.^19^

### Evaluation

Evaluation was considered to be a formal process by which a body of information was systematically gathered and used to make a judgement about the overall value of a teleradiology intervention. Although several papers spoke of ‘evaluation’, none provided a detailed and structured description for a project or programme evaluation.

An ‘evaluation’ for a teleradiology programme training course noted that those actually taking the X-rays but who were not trained radiographers, gained little from the course as they lacked the necessary background knowledge.^17^ Another paper ‘evaluated’ the success of a teleradiology programme by simply observing that it ‘provide[d] care to an underserved population in a cost-efficient way that surpasses the many existing technical, legal and language barriers, leads to improved outcomes and shows promise for sustainability’.^16^

A review of the national teleradiology programme in Mali found that it improved the diagnosis of patients and improved the referring doctor’s ability to give an accurate diagnosis.^18^ Use of the teleradiology service varied widely between hospitals and successful implementation depended on local ownership and close collaboration of stakeholders. A survey conducted as part of the review of the Mali programme identified benefits of teleradiology as better patient diagnosis (91%), improved treatment (30%), opportunity for learning for healthcare personnel (39%), time and cost savings for patients (31%) and better rural healthcare (26%). Challenges were identified as unreliable Internet connections (42%), conflicts (8%), insufficient support from hospital directors (20%) and the cost of the service to patients (12%). It was noted that referral for specialist diagnosis was simply too difficult (up to 1000 km on insecure roads) and prohibitively expensive for many patients (with over 40% of the population living below the international poverty line of $1.25 per day).^18^

Although not a formal evaluation, one study assessed the diagnostic accuracy of digital photographs of plain film chest X-rays (CXRs) obtained using a mobile phone and demonstrated non-inferiority with plain film CXRs. The authors noted this finding had important implications for resource-constrained countries with limited access to radiologists.^22^ Findings from a telemedicine implementation (which included a teleradiology component) concluded there is no perfect ‘one-size-fits-all’ technology and local context and non-technological factors are influential.^20^ Finally, an Angolan study ‘evaluated’ local feasibility of digital radiology by examining implementation difficulties, continued on-site training needs, maintenance difficulties over two years, changes in work volumes over time and X-ray quality (assessed by two external radiologists).^19^

### Costing

Costing was described as evidence of a structured process being undertaken to determine the expense incurred in establishing a teleradiology program. Five papers addressed some aspects of cost,^16,18,19,20,23^ but none used a formal costing (e.g. activity-based costing) or economic evaluation (e.g. cost–benefit analysis) method.

Simple low-cost solutions have been successfully employed although in 2011 and 2015 the capital cost of technology remained a major barrier.^17,18^ One hospital charged patients 1500.00 – 2500.00 West African CFA Franc (approximately $2.00 – $5.00) to cover technician allowance, electricity, Internet connection, technology maintenance and a small payment to the radiologist, whilst a private health clinic charged about $8.00 to a mining company for routine CXRs and emergency procedures.^18^

The opportunity cost of telecom services was noted in an Ethiopian study (competing with basic priorities such as food, clothing and school fees) that made Internet and mobile phones unaffordable although increasing penetration rates of mobile technology in SSA may ease this.^20^ A fourth paper highlighted how donations from faith-based organisations could fund initial setup costs, with an ongoing equipment service contract largely offset by savings on film, processing chemicals, film bags and storage.^23^ This was supported by another paper that noted their initial digital radiology investment (considered ‘much more affordable’ as equipment costs dropped over time) was paid back in two years by eliminating the need for radiographic films and reagents and might even provide a net cost saving in the middle to long term.^19^

### Image display

Papers were examined to determine whether they described the means by which visual presentation of images was achieved. Four papers provided some insights. Importantly, one report noted that ‘several studies’ have shown JPEG images obtained by digital photography of film radiographs, using limited image compression, were sufficient for diagnosis in most instances.^21^ Shiferaw and Zolfo also reported taking photographs of radiographs with a camera, using natural light on a white glared window, produced good-quality images.^20^ Similarly, a third paper reported that diagnostic accuracy of common pathologies and normal findings in CXRs was comparable using a light box for viewing of films compared to photographs taken by a digital camera.^22^ Another paper also noted image visualisation time was reduced to about half when images were viewed on a 1600 × 900 dots per inch (dpi) monitor versus traditional viewing, due to the higher resolution.^19^

### Image capture

For capture, papers were required to describe the means by which images were captured for transfer to or from a device connected to the computer or the network. Eight papers described elements related to this, with noted approaches including the use of digital cameras, document scanners, specialised digitisers or computerised radiography systems that directly produced digital images.^17,18,19,20,21,22,24,25^ Use of digital cameras was common,^16,18,21,22,24^ but only one paper specifically identified the use of a phone-based digital camera.^22^

### Concordance

For concordance, papers were expected to describe formal and focused assessments of the degree of clinical agreement obtained when using teleradiology or mobile teleradiology versus traditional radiology. None of the papers provided any description of formal ‘concordance studies’ although equivalence or diagnostic accuracy was assessed. One study addressed mobile teleradiology and demonstrated non-inferiority of mobile phone digital photographs of CXRs compared to plain film CXRs for some pathologies.^22^ These authors concluded ‘The non-inferiority of mobile teleradiology compared to interpretation of plain films has important implications for health care in resource-poor countries with limited access to radiologists’.

## Discussion

This study has identified that there are currently few published papers reporting national teleradiology networks within SSA despite the repeatedly stated need to establish these services and overcome the sub-continent’s lack of health human resources (including radiologists).^1,20,21^ Other contributing arguments are widespread poor radiologic interpretation and the negative impact on morbidity and mortality of shortages in radiology services.^18^ In 2014, it was reported that shortages in radiology services affected 3.5–4.7 billion people worldwide – about one-half of the global population.^16^ The establishment of teleradiology services, in particular relatively inexpensive mobile teleradiology, in SSA, could mitigate some health systems and healthcare needs. However, there is a lack of comprehensive and published studies to guide the introduction of teleradiology or mobile teleradiology. In particular, none of the selected studies thoroughly addressed needs assessment, implementation, connectivity, evaluation, costing, image display, image capture or concordance in relation to mobile teleradiology.

Surprisingly, only one paper specifically identified the use of a phone-based digital camera, as used for mobile teleradiology.^22^ Typically physicians will use their own smartphone to capture images, and significant advances have been made in recent years with smartphones allowing capture and transmission of high-resolution images in jpeg, png or gif file formats. A limitation is that these file formats do not have steganographic data embedded in them. If needed for storage in a PACS, they could be converted to Digital Imaging and Communications in Medicine (DICOM) but without any embedded data.

Limitations to the study exist. Only two databases were searched: a subject database (PubMed; biomedical literature) and a citation database (Scopus; life sciences, social sciences, physical sciences and health sciences). A further limitation was the *a priori* selection of the eight categories for charting. Additional issues relevant to mobile-teleradiology arose but were not charted. For example, the following were not charted: legal, regulatory and ethical issues; the quality and quantity of information provided at consultation; data security during transmission; data storage and record keeping and patient identification. Such issues must be considered when establishing any clinical programme. Moreover, more teleradiology focussed issues, such as camera and screen resolution, file format and the use of RIS and PACS, were not charted in depth. Although some of the above issues were noted in a few papers (Table 1), they were not addressed or debated in detail in these literature sources, except by Mars.^24^

All of the selected papers justified ‘need’ on the basis of the shortage or absence of radiologists in each setting.^16,17,20,21^ However, services developed without appropriate planning and without clear understanding of community needs, local clinical requirements and available infrastructure may fail to be sustained and/or scaled. Yet there is little guidance on practical ‘needs assessment’.^27^ For example, satisfactory connectivity must be demonstrated and affordable whether through wired or wireless Internet or cellular networks or both.^20^ Similarly, a reliable electricity supply is desirable^9,17,20,21^ but is a longstanding issue in SSA that negatively impacts healthcare delivery.^28,29^ For mobile teleradiology, smartphone use must be available and a reliable cellular network infrastructure capable of inexpensively providing real-time connectivity is a prerequisite. Increasingly, both are the case in SSA.^20,28,29,30,31^

Despite the above, the papers did show that various teleradiology solutions, including mobile teleradiology, are feasible and satisfactory for most routine X-ray needs. Conventional X-ray radiography (with attendant shortcomings: controlled temperature and humidity, water use, chemical use, incomplete drying and films sticking together^17^) is still one of the most widely used medical diagnostic techniques in rural healthcare centres in the developing world. These locations do not usually have radiologists on hand, making teleradiology a useful tool. This includes mobile teleradiology where smartphones with digital cameras are used to capture plain film images for transmission to and interpretation by consultant radiologists at a distance.^22^ Leveraging teleradiology, in particular mobile teleradiology, could mitigate the shortage of radiologists outside major centres, improve patient diagnosis and care, reduce the travel burden for patients and do so inexpensively^18,27^ but requires local buy-in, multi-sectoral support and capacity building.^20^ Of interest was the suggestion that the primary role of teleradiology should be facilitating training and capacity building and not consultation^19^ and that reliance on external resources or North-South collaborations for support should be reduced through local training and capacity building.^16^ Certainly, radiology training varies significantly between countries in Africa,^32^ and teleradiology training and skill building could, with some provisos, be mediated through technology.^33,34^

Available literature from outside SSA indicates diagnostic imaging reduces mortality and improves the quality of life, is necessary for global health and teleradiology improves specialist access and reduces travel time and cost for patients.^35,36,37,38^ Given that decision- and policy-makers need local evidence to inform their decisions, and based on the findings of this review, it is recommended that:

- A small number of well-designed and comprehensive studies be performed in SSA to confirm the utility and cost of mobile teleradiology versus conventional radiology and provide clear evidence-based guidelines for implementing mobile teleradiology programmes.
- These studies are published in open-access journals to ensure free availability to the developing world, in particular SSA.
- Given that any e-health solution is an opportunity cost and (when competing with vaccination, safe water supply and other primary healthcare priorities) must fill a justified and compelling need, any rural or remote healthcare facility where mobile teleradiology is considered must perform a local needs assessment to confirm projected viability.
- Where viability is determined, mobile teleradiology should be phased in with appropriate training and support.

## Conclusion

Teleradiology, the transmission of radiological patient images (X-rays, CTs, MRIs) from one location to another in order to share these studies with other clinicians for consultation or interpretation, is considered one of the oldest, most established, successful and widely used clinical telemedicine specialties. This study confirms that improvements in device and information technology have enabled advances in teleradiology and also opened the field of mobile teleradiology. With contemporary smartphones and mobile networks, technical issues such as image quality, transmission speed and image compression are no longer major barriers although issues such as adequate and reliable local connectivity and electricity remain.

It has also shown that teleradiology, including the use of mobile teleradiology, is simple, feasible and appropriate for routine X-ray support of patients and healthcare providers in rural and remote locations, but woefully underused in SSA, although regional evidence to adequately demonstrate this is limited. Nonetheless, teleradiology has proven benefits for healthcare delivery (e.g. improved referring doctor’s diagnostic accuracy) and patient care (e.g. reduced travel and associated expense) and offers a solution to settings where there are few or no radiologists. The almost routine application of teleradiology and mobile teleradiology for routine imaging tasks elsewhere reflects the changing world of clinical practice, service delivery and technology.

The limited mobile teleradiology in SSA, as evidenced in the literature, hampers patient access to basic essential radiological services. There is an urgent need for rigorous published research that will produce high quality and evidence-based guidance for the establishment of mobile teleradiology programmes in SSA. This would both align with the World Health Organization’s (WHOs) support of mhealth, the achievement of universal health coverage (UHC) and the United Nation’s sustainable development goals (SDGs),^39^ as well as support Nigeria’s renewed interest in digital health.^40^ Successful programmes are likely to be one aspect of a holistic national ehealth strategy that will guide innovation to address evidence-based needs and result in broader user adoption. Furthermore, successful implementation of teleradiology may encourage more widespread and successful adoption of other e-health solutions.
